# Mental disorders in internally displaced persons: clinical features, therapy, prevention

**DOI:** 10.1192/j.eurpsy.2024.369

**Published:** 2024-08-27

**Authors:** N. O. Maruta, T. V. Panko, V. Y. Fedchenko, I. O. Yavdak, O. E. Semikina

**Affiliations:** Borderline psychiatry, “Institute of Neurology, Psychiatry and Narcology of the NAMS of Ukraine” SI, Kharkiv, Ukraine

## Abstract

**Introduction:**

Military aggression significantly worsens the mental health of the population due to losses: human, economic-financial, social-psychological, ecological. One of the negative consequences of military aggression is migration, the movement of the population within the country and outside its borders, which disrupts adaptation and leads to the formation of a wide range of psychological and mental disorders.

**Objectives:**

To study the state of mental health of internally displaced persons (IDPs) and to develop, on this basis, a system of therapy and prevention of mental disorders.

**Methods:**

A combination of clinical-psychopathological and psychodiagnostics methods of studying were used to identify peculiarities of the mental state and various forms of mental pathology.

**Results:**

270 IDPs were examined. It was established that the group of IDPs is heterogeneous and includes 3 categories: 1 group of IDPs who do not have signs of mental disorders (conditionally healthy) (34.81%), 2 group of IDPs who have separate symptoms of mental disorders (risk group) (25.19 %) and the 3 group of IDPs with established mental disorders (40.00 %). The assessment of the general condition of the IDPs of the 1 group shows that they have various somato-neurological disorders and the absence of mental disorders. The assessment of the emotional state of the IDPs of the 2 group indicates the presence of various emotional disorders that include asthenic syndrome (41.18 ± 5.97) %, agripnic syndrome (45.59 ± 6.04) %, somato-vegetative syndrom (30.88 ± 5.60) %, anxiety-depressive syndrome (20.59 ± 4.90) %). That separate syndromеs do not meet the criteria of ICD-10 and were subthreshold. The general features of mental disorders in group 3 IDPs are characterized by the predominance of disorders of the anxiety-depressive spectrum and includes adjustment disorders (F43.2) in 35,18%; post-traumatic stress disorder (F43.1) in 17.59%; a moderate depressive episode (F32.1) in 16, 67%; organic affective (depressive) disorder (F06.3) in 14.81%; organic anxiety disorder (F06.4) in 9.28%; recurrent depressive disorder (33.1) in 6.48%.

**Conclusions:**

A system of treatment of mental disorders in IDPs has been developed, which includes pharmacotherapy (the usage of antidepressants, anxiolytics and, if necessary, antipsychotics), cognitive-behavioral and family psychotherapy. For prevention mental disorders among IDPs of groups 1 and 2, a psychoeducational program was created, aimed at forming awareness of clinical manifestations of mental disorders, opportunities to prevent their formation, and necessary actions in conditions of exacerbation of the mental state. Evaluation of the effectiveness of the developed system of therapy and psychoeducation has testified to their effectiveness.

**Disclosure of Interest:**

None Declared

